# The mediating role of children’s intergenerational support in association between grandparenting and cognitive function among middle-aged and older Chinese: findings from the CHARLS cohort study

**DOI:** 10.1186/s12889-024-18106-8

**Published:** 2024-02-23

**Authors:** Xuebei Hou, Yujun Luo, Fen Yang, Xinhong Zhu, Xiaolian Gao, Wenqiang Wang, Guiyuan Qiao, Jing Zhou

**Affiliations:** 1 School of Nursing, Hubei University of Chinese Medicine, Wuhan, China; 2 Department of Tuina and Rehabilitation Medicine, Hubei Provincial Hospital of Traditional Chinese Medicine, Wuhan, China; 3 Department of Tuina and Rehabilitation Medicine, Affiliated Hospital of Hubei University of Chinese Medicine, Wuhan, China; 4 Department of Tuina and Rehabilitation Medicine, Hubei Provincial Institute of Traditional Chinese Medicine, Wuhan, China; 5 First Clinical Medical College, Hubei University of Chinese Medicine, Wuhan, China

**Keywords:** Grandparenting, Cognitive function, Children’s intergenerational support, Cohort study, CHARLS

## Abstract

**Objectives:**

With the world’s population increasing in age, there has been a significant rise in the prevalence of cognitive impairment and dementia among individuals. This study aims to investigate the association between grandparenting and cognitive function among middle-aged and older Chinese using data from 2011 to 2018 China Health and Retirement Longitudinal Study (CHARLS). Additionally, the study seeks to explore the potential mediating effect of intergenerational support from children on this relationship, using data from the CHARLS 2011 database.

**Methods:**

5254 participants were recruited at the baseline survey in CHARLS 2011. Subsequently, a follow-up survey was conducted over 8 years, from CHARLS 2011 to 2018, with 1472 individuals completing the follow-up survey. The CHARLS included surveys on grandparenting and cognitive assessments. Grandparenting was categorized as yes and no. The assessment of cognitive function involved the evaluation of episodic memory and mental intactness. The present study used cross-sectional and longitudinal analyses to examine the relationship between grandparenting and cognitive function. The bootstrap method assessed the mediating effect of children’s intergenerational support.

**Results:**

The results of both cross-sectional and longitudinal studies indicated a positive association between grandparenting and cognitive function in middle-aged and older Chinese (B = 0.138, *p* < 0.05; B = 0.218, *p* < 0.05). Children’s emotional and economic support played intermediary roles between grandparenting and cognitive function.

**Conclusion:**

The results emphasized the significance of policymakers considering the consequences of intergenerational care and family support when formulating and executing social service policies targeted at the middle-aged and older population in China.

## Introduction

As the world’s population increases in age [1], there has been a significant rise in the prevalence of cognitive impairment and dementia among individuals [2]. Worldwide, around 55 million people live with dementia, which is expected to rise to 78 million in 2030 and 139 million in 2050 [3]. It has physical, psychological, social, and economic impacts, not only for people living with dementia but also for their caregivers, families, and society at large [4–6]. Currently, there is no effective treatment for cognitive impairment or dementia [7]. Cognitive impairment and dementia have become significant and increased global health challenges [8]. An urgent need is to find a widespread and cost-effective way to address this issue. In many developing countries, family support remains the primary source of care and support for older people [9]. Especially in China, family care for older adults has always been the most crucial way of supporting the older adults [10]. Hence, it is imperative to reevaluate the significance and role of the family unit and offer governmental assistance to address the requirements of the older population in China effectively.

Intergenerational care is a significant mode of engagement in family support of middle-aged and older adults in China [10]. As for the research on the relationship between intergenerational care and middle-aged and older people, most scholars adopt role strain theory or role enhancement theory to explore the impact of intergenerational care on the cognitive function of middle-aged and older people. According to the role enhancement theory, individuals can achieve social integration and self-fulfillment through engaging in multiple roles, thus positively impacting the individual [11, 12]. Caring for grandchildren enables grandparents to take on more social roles, increasing the interaction between middle-aged and older people and their family members, reducing their loneliness [13], improving their social integration, and bringing higher life satisfaction and self-efficacy [14]. Many studies from Western countries have provided evidence of the positive impact of caring for grandchildren on their cognitive function [15–18]. Moreover, a longitudinal study of older Koreans found that caring for grandchildren could improve global cognitive functioning scores by 30.05% [19]. According to a cross-sectional study of 7704 Chinese adults aged ≥ 45 years from the China Health and Retirement Longitudinal Study (CHARLS), providing moderate, not regular grandparenting or caring for one grandchild was more positively associated with cognitive function [20]. Wang and Zhang also noted that the positive impact of intergenerational care on cognitive function in middle-aged and older adults is more pronounced in men than in women [21]. Additionally, a study based on “the well-being of elderly in Anhui province” 2001–2012 longitudinal survey exploring the impact of providing intergenerational care on grandparents’ cognitive function trajectories, the finding shows that the provision of high-intensity intergenerational care had a significant positive effect on men’s cognitive function [22]. Nevertheless, role strain theory suggests that when role obligations exceed the physical and mental capacity of the individual, it will increase role strain, which is harmful to health [23]. Caring for grandchildren increases the physical burden and reduces self-care in older adults, resulting in cognitive decline [24]. Arpino and Bordone found that providing intensive care for grandchildren harms cognition [25]. Findings from a longitudinal prospective study of 186 Australian women from the Women’s Healthy Aging Project (WHAP) demonstrated that taking care of grandchildren five days a week or more can harm cognitive function [24]. Possible reasons for the mixed results include different analytical strategies used in the statistical analyses, different intensities of care, and socio-cultural contexts [26]. Research on how caring for grandchildren affects grandparents’ cognitive function in China is limited in the literature. Existing studies focus on cross-sectional data or regional data for a particular area for empirical analyses [20–22], and their results may be biased by reverse causality and simultaneity bias or suffer from the disadvantage of being under-represented. Therefore, this study uses longitudinal and large-scale national data to examine the effects of caring for grandchildren on grandparents’ cognitive function in China, thus mitigating the bias caused by the endogeneity of reverse causality and expanding previous studies focusing on cross-sectional or specific regional data.

In China’s aging society and the limited availability of long-term care services for older individuals [27], implementing intergenerational care programs enhances interaction between older adults and their families. Intergenerational support from their offspring is crucial for encouraging active aging among older individuals [28]. Empirical evidence suggests that intergenerational support from adult children serves as a significant mediator in the relationship between changes in the social dimension (such as social support, social interaction, family adaptability, cohesion, and interactions with family and friends) [29] and the physical and mental well-being of older individuals [30, 31]. According to intergenerational exchange theory, caring for grandchildren increases the likelihood of older people receiving more intergenerational support from their adult children [32]. The data from Class 2012 suggested that caring for grandchildren can significantly increase the financial support and the frequency of household work and visits by adult children [33]. Some studies have indicated that emotional and financial support from adult children could reduce the risk of cognitive decline in older adults [30, 34]. Moreover, most studies only explored the direct relationship between caring for grandchildren and cognitive function [20–22], and did not study the underlying mechanism. This study uses children’s intergenerational support as the intermediary variable to conduct path analysis to explore further the mechanisms underlying the effects of grandparenting on cognitive function in middle-aged and older adults.

Given this, we aimed to conduct a longitudinal study using data from 2011 to 2018 China Health and Retirement Longitudinal Study (CHARLS) to examine the relationship between grandparenting and cognitive function of middle-aged and older Chinese. Additionally, the study seeks to explore the potential mediating effect of intergenerational support from children on this relationship, using data from the CHARLS 2011 database. Specifically, we aimed to investigate the following three hypotheses. Hypothesis 1: We hypothesized that grandparenting positively affects the cognitive function of middle-aged and older Chinese. Hypothesis 2: We hypothesized that grandparenting negatively affects the cognitive function of middle-aged and older Chinese. Hypothesis 3: Children’s intergenerational support, including emotional and financial support, may mediate the relationship between grandparenting and cognitive function among middle-aged and older Chinese.

## Methods

### Participants and setting

Data are extracted from the China Health and Retirement Longitudinal Study (CHARLS), an ongoing nationally representative survey in China. The CHARLS was conducted in 2011 (wave 1) and includes 17,705 Chinese residents aged 45 and over from 28 provinces. The sampling technique employed for participant selection was a multistage probability-proportional-to-size (PPS) approach. The CHARLS assessments include community residents’ social, economic, and health circumstances. Consequently, three subsequent assessments were conducted in 2013 (Waves 2), 2015 (Waves 3), and 2018 (Waves 4). The CHARLS survey has obtained ethical approval from the Ethical Review Committee of Beijing University, and all the respondents have signed informed consent forms [35].

**Fig. 1:**
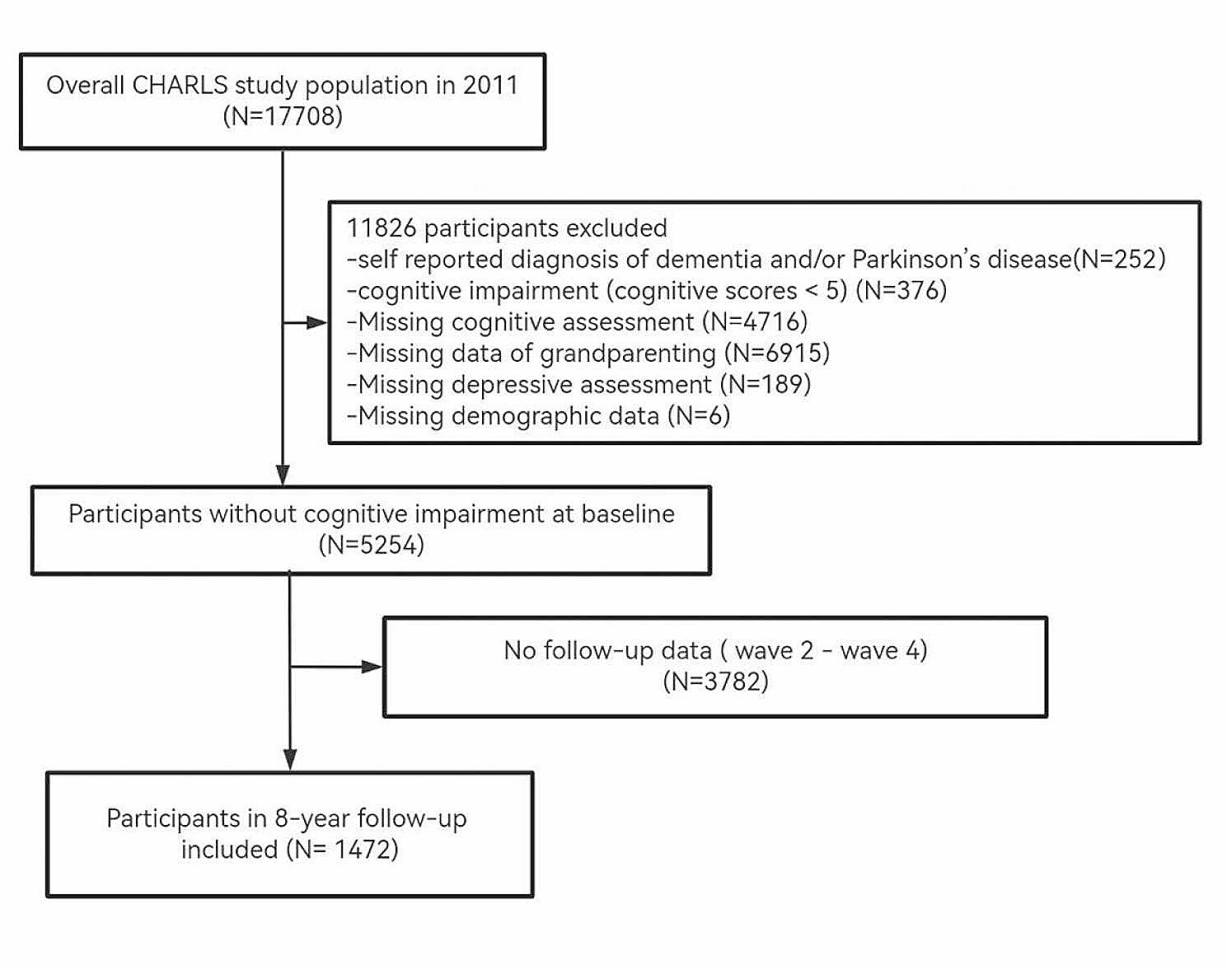
Flowchart of participant selection from the China Health and Retirement Longitudinal Study (CHARLS)

The present analysis utilizes data from four waves (2011, 2013, 2015, and 2018) of the China Health Retirement Longitudinal Study (CHARLS). We restrict individuals who met any of the following criteria at baseline (CHARLS 2011): (1) age ≥ 45 years at baseline; (2) complete the questionnaire about grandparenting; (3) complete cognitive assessments; (4) they have no self-reported diagnosis of dementia or Parkinson’s disease; (5) we exclude individuals have cognitive impairment with baseline cognitive scores < 5 points [1.5 SD below its mean] [36, 37]. Furthermore, we eliminated participants who did not have follow-up data from Wave 2 to Wave 4. A total of 5254 participants completed the baseline survey, and 1472 individuals completed the follow-up survey over eight years (see Fig. 1).

### Measures

#### Grandparenting

The following question is used to measure grandparenting in the CHARLS questionnaire: During last year, did you spend time taking care of your grandchildren? We allocated 1 to the respondents who answered “yes” and 0 to those who answered “no.”

#### Cognitive function

According to previous studies [36, 38], cognitive function is measured by two dimensions: episodic memory and mental intactness. Episodic memory is assessed using word recall tests. In the word recall tests, examiners read a list of 10 Chinese nouns, and respondents are instructed to repeat the words in any order immediately (immediate recall) and to recall the same list of words 4 min later (delayed recall). The episodic memory score is calculated as the average of the immediate and delayed recall scores, ranging from 0 to 10. Mental intactness is evaluated by ten items from the Telephone Interview of Cognitive Status test (TICS-10) [39] and a pentagon-drawing test. In CHARLS, mental status questions include serial subtraction of 7 from 100 (up to 5 times), date (month, day, year), day of the week, season, and intersecting pentagon copying test for 11 points. Total cognitive scores are calculated as the sum of episodic memory and mental intactness scores and range from 0 to 21, with higher scores indicating better cognitive function [40].

#### Children’s intergenerational support

Children’s intergenerational support is measured from emotional and financial support [32]. In CHARLS, children’s economic support is measured by asking the respondent about the total amount of money and goods received from children in the past year. In order to avoid the influence of extreme values, the value of this variable is added"1” and then logarithm (that is, ln(x + 1)) in the empirical analysis of this paper. According to the CHARLS questionnaire, children’s emotional support is measured by the frequency of contact with parents: “How often do you communicate with children you do not live with?” According to a previous study [32], the ten options of the questionnaire are reclassified into five options, which are “hardly,” “seldom,” “almost monthly,” “almost weekly,” and “almost every day,” and assigned a value of 0–4 in turn. In addition, we assign a value of 5 if respondents are living with their children. The total contact frequency score is 5, with higher scores indicating higher contact frequency with their children.

#### Covariates

The socio-demographic information is obtained from the baseline survey, including age (≥ 45 years), gender (female = 0, male = 1), education level (illiterate = 0; primary school = 1; middle school = 2; high school or above = 3), residence (urban = 0; rural = 1). Activities of daily living (ADLs) are measured by whether the respondent has difficulty in dressing, bathing and showering, eating, getting in and out of bed, using the toilet, and controlling urination and defecation. As long as one item is difficult, it is scored 1, and all six items with no difficulty are counted as 0. Depressive symptoms are measured by the 10-item Center for Epidemiologic Studies Depression Scale (CES-D), which has been validated and widely used among Chinese adults [41]. The CESD-10 consists of 10 items: (1) felt depressed, (2) bothered by little things, (3) had trouble concentrating, (4) sleep was restless, (5) everything was an effort, (6) felt hopeful, (7) felt happy, (8) felt fearful, (9) could not get going, and (10) felt lonely. Each item uses a four-point Likert scale. The four options are “rarely or none of the time,” “some or a little of the time,” “occasionally or a moderate amount of the time, “and “most or all of the time.” Negative symptoms are assigned 0, 1, 2, and 3. On the contrary, two positive symptoms take values of 3, 2, 1, and 0. The total score on the scale is between 0 and 30 points, with the higher scores indicating more depressive symptoms.

### Statistical analysis

Descriptive statistics are used to present the baseline characteristics of the enrolled participants. Continuous variables are expressed using mean and standard deviation (SD), while percentages are used for categorical variables. Chi-square tests and independent sample T-test are performed to compare the differences between participants with and without grandchildren caregiving at the baseline survey. The Kruskal-Wallis test is performed to compare the cognitive function differences among four data waves.

In this study, we estimate two regression models: cross-sectional and 8-year follow-up models. Multivariate linear regression analysis is performed in the cross-sectional model. The generalized linear mixed model (GLMM) provides a valuable approach for analyzing longitudinal data in an 8-year follow-up model [42].

The PROCESS macro for SPSS 25 is used to conduct the mediation analysis to investigate whether children’s intergenerational support mediates the effect of grandparenting on cognitive function at baseline. The mediating effect is tested using a bootstrap estimation approach with 5000 repetitions. The indirect effect is significant when the 95% CI does not contain 0.

All statistical analyses are performed with the IBM SPSS Statistics version 25. *P* < 0.05 is statistically significant.

## Results

### Baseline characteristics

Table 1 shows the baseline characteristics of the 5254 participants. The mean (SD) age at baseline was 58.88 (7.78) years. The average total cognitive score was 11.32 (3.37), and the average for depressive symptoms was 8.46 (6.25). Of all participants, most of them lived in rural areas (79.0%), had a primary school educational level (47.2%), had no difficulty in activities of daily living (85.6%), and had weekly contact with children (36.5%). For the entire sample, 49.5% of participants were involved in taking care of grandchildren. Moreover, there was a significant difference in socio-demographic characteristics, cognitive function, depressive symptoms, ADLs, and contact frequency with children between non-caregivers and caregivers (*P* < 0.05). Compared with participants who did not provide grandchildren care, those who cared for grandchildren had lower depressive symptoms scores and higher total cognitive scores.

**Table 1:** Baseline characteristics of participants

Variable	Total	Caring for grandchildren		*P* value
	*N* = 5254	No N(%) 2652(50.5)	Yes N(%) 2602(49.5)	
Continuous variables mean(SD)				
Cognitive function	11.32(3.37)	11.12(3.35)	11.57(3.37)	< 0.001
Age	58.88(7.78)	59.96(8.51)	57.77(6.79)	< 0.001
Depressive symptoms	8.46(6.25)	8.73(6.37)	8.19(6.11)	< 0.001
Ln(financial support)	2.90(3.75)	2.85(3,71)	2.96(3.79)	0.263
Categorical variables N(%)				
Gender				0.033
Female	2531(48.2)	1239(23.6)	1292(24.6)	
Male	2723(51.8)	1413(26.9)	1310(24.9)	
Educational level				0.049
Illiterate	1085(20.7)	588(11.2)	497(9.5)	
Primary school	2482(47.2)	1233(23.5)	1249(23.8)	
Middle school	1108(21.1)	541(10.3)	567(10.8)	
High school or above	579(11.0)	290(5.5)	289(5.5)	
Residence				< 0.001
Urban	1105(21.0)	500(9.5)	605(11.5)	
Rural	4149(79.0)	2152(41.0)	1997(38.0)	
Activities of Daily Living				0.017
No	4498(85.6)	2240(42.6)	2258(43.0)	
Yes	412(14.4)	344(7.8)	344(6.5)	
Contact Frequency				< 0.001
Seldom	552(10.5)	338(6.4)	214(4.1)	
Hardly	176(3.3)	102(1.9)	74(1.4)	
Almost every month	1103(21.0)	587(11.2)	516(9.8)	
Almost every week	1918(36.5)	1013(19.3)	905(17.2)	
Almost every day	838(15.9)	395(7.5)	443(8.4)	
Living together	667(12.7)	217(4.1)	450(8.6)	

Table 2 shows a significant difference in cognitive function among four waves. The mean score of participants’ cognitive function was 11.37(3.46), 11.17(3.79), 10.68(3.93), and 10.80(4.80) during wave 1,2,3, and 4, respectively.

**Table 2:** Descriptive statistics for cognitive function scores from 2011 to 2018 among middle-aged and older adults in China (N = 1427)

Setting	2011 Wave 1	2013 Wave 2	2015 Wave 3	2018 Wave 4	*P* value
Cognitive function(0–21) mean(SD)	11.37(3.46)	11.17(3.79)	10.68(3.93)	10.80(4.80)	0.003

Table 3 reports the results of cross-sectional and 8-year follow-up models of the relationship between grandparenting and cognitive function of middle-aged and older adults. As shown in model 1, the results of the cross-sectional model showed that grandparenting had a significant positive association with the cognitive function of middle-aged and older adults (B = 0.138, *p* < 0.05). The result of the 8-year follow-up model was consistent with the cross-sectional model, suggesting a favorable cognition impact of grandchildren care (B = 0.218, *p* < 0.05). In addition, other control variables also had a significant impact on the cognitive function. Children’s emotional and economic support and educational level significantly positively affected the cognitive function of middle-aged and older adults. In contrast, rural residents, ADLs and depressive symptoms negatively affected the cognitive function of middle-aged and older adults.

**Table 3:** Association between grandparenting and cognitive function in middle-aged and older adults

Cognitive function	Model 1 Cross-sectional (*N* = 5254)		Model 2 8-year follow-up (*N* = 1472)
	B (95% CI)		B (95% CI)
Caring for grandchildren (Reference: No)	0.138 (0.014, 0.342)*		0.218 (0.041, 0.394)*
Gender (Reference: Female)			
Male	0.342 (0.156, 0.519)***		0.740 (0.550, 0.930)***
Age	-0.025 (-0.036, -0.014)***		-0.062(-0.074, -0.050)***
Educational level (Reference: Illiterate)			
Primary school	1.193 (1.709, 2.153)***		2.544 (2.305, 2.783)***
Middle school	3.158 (2.889, 3.427)***		3.692 (3.398, 3.985)***
High school or above	3.885(3.559, 4.212)***		4.433(4.100, 4.766)***
Residence (Reference: Urban)			
Rural	-0.889(-1.102, -0.677)***		-1.378(-1.629, -1.126)***
ADLs (Reference: No)	-0.615(-0.855, -0.375)***		-0.238(-0.471, -0.006)*
Depressive symptoms	-0.087(-0.100, -0.073)***		-0.090(-0.104, -0.076)***
Financial support	0.029(0.007, 0.051)*		0.048(0.024, 0.072)***
Emotional support	0.107(0.046, 0.167)**		0.079(0.016, 0.142)*

Note: Model 1: Outcomes of multivariate linear regression; Model 2: Outcomes of generalized linear mixed model (GLMM)

*** *p* < 0.001, ** *p* < 0.01, * *p* < 0.05

### Mediation effect test

**Fig. 2:**
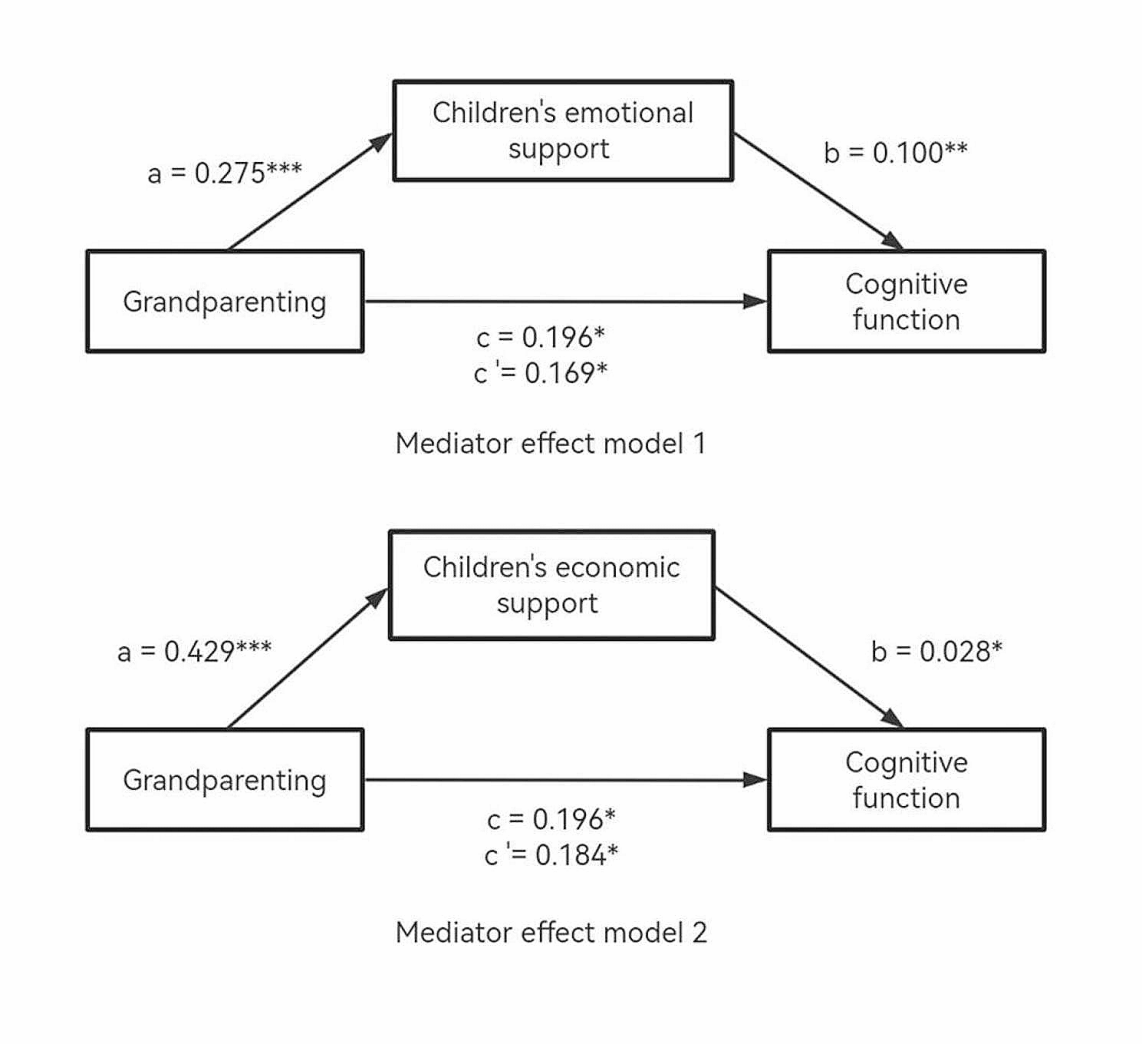
The conceptional framework of the mediation models *Note*: All models were adjusted for age, gender, educational level, residence, depressive symptoms, and ADLs * *p* < 0.05, ** *p* < 0.01, *** *p* < 0.001

Table 4 provides the results of mediation analyses. Figure 2 demonstrates the model process of children’s intergenerational support mediated the relationship of grandparenting with the cognitive function of middle-aged and older adults. As shown in Table 4, model 1, the upper and lower bounds of the bootstrap 95% confidence interval of the children’s emotional support’s indirect effect did not contain 0, so it was considered that there was a mediating effect [43]. Similar results were observed for model 2, the confidence interval of the children’s economic support’s indirect effect did not contain 0, and there was also a mediating effect.

For Fig. 2, model 1, after controlling for age, gender, and other potential confounders, path a showed that grandparenting had a significant positive effect on children’s emotional support (B = 0.275, *p* < 0.001). Path b showed that children’s emotional support significantly improved cognitive function (B = 0.100, *p* < 0.01). Path c was the total effect of grandparenting responding primarily to cognition (B = 0.196, *p* < 0.05), and c’ displayed the positive effect of grandparenting on cognitive function, including the mediating children’s emotional support (B = 0.169, *p* < 0.05). The mediating effect (indirect effect, ab = c–c’) of children’s emotional support was 0.027.

As shown in Fig. 2, model 2, path a showed that grandparenting had a significant positive effect on children’s economic support (B = 0.429, *p* < 0.001). Path b showed that children’s economic support significantly improved cognitive function (B = 0.028, *p* < 0.05). Path c represented the link between grandparenting and cognitive function (total effect). When the children’s economic support is included as an intermediary variable in the model, the coefficient of total effect was reduced to c’ (direct effect). The mediating effect (indirect effect) of children’s economic support was 0.012.

**Table 4:** Analysis of mediation effect

Pathway	Effect	bootsSE	BootLLCI	BootULCI
Model 1				
Direct effect	0.169	0.833	0.006	0.332
Indirect effect	0.027	0.010	0.010	0.047
Model 2				
Direct effect	0.184	0.083	0.021	0.347
Indirect effect	0.012	0.007	0.003	0.024

## Discussion

This study examined how grandparents’ cognitive function was associated with grandparenting using a nationwide longitudinal survey in China. The results suggested that grandparenting had a beneficial effect on grandparents’ cognition. Moreover, children’s intergenerational support mediated the relationship between grandparenting and cognitive function among middle-aged and older Chinese.

In this study, the finding on the positive correlation between grandparenting and cognitive function among middle-aged and older adults is consistent with the previous study and supports the role enhancement theory. Hypothesis 1 was verified. As older people age and lose their previous social roles, caring for grandchildren becomes vital compensation for social role deficits in old age [10]. The social role of taking care of grandchildren increases opportunities for interaction with grandchildren, alleviates emotional deficits in middle-aged and older adults, and improves well-being, which may mitigate depression-related effects and contribute to maintaining cognitive function status [44, 45]. Moreover, caring for grandchildren is an essential form of social engagement [17]. Providing care for grandchildren can enhance grandparents’ daily activities, such as engaging in a wider variety of activities with grandchildren and taking on the responsibility of caring for grandchildren’s daily needs, making them feel youthful and energetic, thereby enhancing social participation and benefiting their cognitive function [46–48]. In addition, caring for grandchildren is a combination of physical and mental work, such as assisting with school or homework, instructing and teaching grandchildren skills, which helps to enhance their mental stimulation by exercising their cognitive skills such as learning, thinking, and reasoning, thus improving cognitive function [49, 50].

Children’s intergenerational support is significantly positively correlated with the cognitive function of middle-aged and older adults, consistent with the results of previous studies [30, 34]. This study showed that the children’s intergenerational support mediated the relationship between grandparenting and cognitive function of middle-aged and older adults. Caring for grandchildren indirectly affects cognitive function in middle-aged and older adults by obtaining intergenerational support from children. Hypothesis 3 was verified. Previous research has found that increasing communication between two generations helps older adults better adjust to stressful events, reduces the risks of depression, and improves their cognitive function [30, 51, 52]. Children’s financial support will be helpful to relieve the stress of financial hardship and enhance the quality of life of older adults, thereby mitigating the stress-related impact on cognition [53, 54]. Caring for grandchildren is crucial to gaining intergenerational support from adult children [10]. From the perspective of intergenerational exchange, older parents work very hard to care for their grandchildren, and their adult children rightly provide support as an exchange of resources [55]. Generally speaking, caring for grandchildren not only frees adult children from the arduous labor of caring for minor children and increases family wealth, but it also strengthens the intergenerational bond between older adults and their children, enabling older adults to receive more financial and emotional support from their adult children, which can have a positive impact on older adults and contribute to their cognitive function [30, 51–55]. This exchange relationship is a two-way intergenerational support between parents and children, corresponding to the intergenerational exchanges in the family in the form of “upbringing support” [56].

There is a growing trend of increased female participation in social labor, but the social support for child-rearing is seriously inadequate in China [57]. The significance and worth of intergenerational care must be considered. It is imperative for society and families to actively promote the involvement of older individuals in the care of their grandchildren. This practice reduces the caregiving burden on the parents and contributes to societal advancement. Simultaneously, adult children must provide more intergenerational assistance to the older individuals who actively care for their grandchildren to alleviate the mental and psychological burdens associated with caregiving. With calls for healthy aging, there is a need to improve the quality of intergenerational relationships and establish positive intergenerational exchange patterns. Society and families must prioritize the older population’s healthcare concerns and living requirements. In order to promote intergenerational communication, it is recommended that the government implement policies aimed at enhancing family leave provisions and allocating additional time for intergenerational interaction [52].

The present study has certain limitations. First, our measure of caring for grandchildren is a binary variable, indicating ‘yes’ or ‘no’. We do not consider the number of grandchildren cared for and the intensity of grandchild care. Secondly, this paper does not control the grandchildren’s ages due to data limitations. The care needs of grandchildren vary considerably by age [58]. Finally, cognition evaluation is subjective and unstable. There is no clinical diagnosis of cognitive impairment.

## Conclusion

The results of the present study revealed that grandparenting was significantly positively related to the cognitive function of middle-aged and older Chinese, and children’s intergenerational support mediated the relationship between grandparenting and cognitive function. Therefore, policymakers must consider the consequences of intergenerational care and family support when formulating and executing social service policies targeted at the middle-aged and older population in China.

## Data Availability

The database (CHARLS), which is used in this paper, is publicly available. (http://charls.pku.edu.cn/en).
